# Which government policies to create sustainable food systems have the potential to simultaneously address undernutrition, obesity and environmental sustainability?

**DOI:** 10.1186/s12992-024-01060-w

**Published:** 2024-07-27

**Authors:** Celia Burgaz, Iris Van-Dam, Kelly Garton, Boyd A. Swinburn, Gary Sacks, Gershim Asiki, Rafael Claro, Adama Diouf, Ana Paula Bartoletto Martins, Stefanie Vandevijvere

**Affiliations:** 1 Department of Epidemiology and Public Health, Sciensano, Brussels, Belgium; 2 Department of Geosciences, Environment and Society, Free University of Brussels (ULB), Brussels, Belgium; 3 School of Population Health, University of Auckland, Auckland, New Zealand; 4 Global Centre for Preventive Health and Nutrition (GLOBE), Deakin University, Melbourne, Australia; 5 Chronic Diseases Management Unit, African Population and Health Research Center, Nairobi, Kenya; 6 Nutrition Department, Universidade Federal de Minas Gerais, Belo Horizonte, Minas Gerais, Brazil; 7 Laboratoire de Recherche en Nutrition Alimentation Humaine, Université Cheikh Anta Diop, Dakar, Senegal; 8 Nutrition Department, School of Public Health, São Paulo University, São Paulo, Brazil

## Abstract

**Introduction:**

A transformation of food systems is urgently needed, given their contribution to three ongoing and interlinked global health pandemics: (1) undernutrition and food insecurity, (2) obesity and non-communicable diseases (NCDs), and (3) climate change and biodiversity loss. As policymakers make decisions that shape food systems, this study aimed to identify and prioritise policies with double- or triple-duty potential to achieve healthier and more environmentally sustainable food systems.

**Methods:**

This study undertook a 4-step methodological approach, including (i) a compilation of international policy recommendations, (ii) an online survey, (iii) four regional workshops with international experts and (iv) a ranking for prioritisation. Policies were identified and prioritised based on their double- or triple-duty potential, synergies and trade-offs. Using participatory and transdisciplinary approaches, policies were identified to have double- or triple-duty potential if they were deemed effective in tackling two or three of the primary outcomes of interest: (1) undernutrition, (2) obesity/NCDs and (3) environmental degradation.

**Results:**

The desk review identified 291 recommendations for governments, which were merged and classified into 46 initially proposed policies. Based on the results from the online survey, 61% of those policies were perceived to have double- or triple-duty potential. During the workshops, 4 potential synergies and 31 trade-offs of these policies were identified. The final list of 44 proposed policies for healthier and more environmentally sustainable food systems created was divided into two main policy domains: ‘food supply chains’ and ‘food environments’. The outcome with the most trade-offs identified was ‘undernutrition’, followed by ‘environmental sustainability’, and ‘obesity/NCDs’. Of the top five expert-ranked food supply chain policies, two were perceived to have triple-duty potential: (a) incentives for crop diversification; (b) support for start-ups, and small- and medium-sized enterprises. For food environments, three of the top five ranked policies had perceived triple-duty potential: (a) affordability of healthier and more sustainable diets; (b) subsidies for healthier and more sustainable foods; (c) restrictions on children's exposure to marketing through all media.

**Conclusion:**

This study identified and prioritised a comprehensive list of double- and triple-duty government policies for creating healthier and more environmentally sustainable food systems. As some proposed policies may have trade-offs across outcomes, they should be carefully contextualised, designed, implemented and monitored.

**Supplementary Information:**

The online version contains supplementary material available at 10.1186/s12992-024-01060-w.

## Introduction

Food systems are the complex and interconnected range of actors and their interlinked value-adding activities involved in the production, aggregation, processing, distribution, consumption and disposal of food products that originate from agriculture, forestry or fisheries, and parts of the broader economic, societal and natural environments in which they are embedded [22]. The term also includes the inputs needed, and the outputs generated, at each of these steps [11]. Our current food systems are under scrutiny: ever since the Green Revolution in the 1950s, agricultural innovations and technologies have managed and evolved to feed a fast-growing population with an abundance of low-cost food [15]. However, the primary focus of the regulation of food systems remains on food quantity and economic benefits to suppliers, often at the expense of quality and ecology. Such a regulatory focus has contributed to unhealthy, environmentally unsustainable and socially unjust food systems across the world [3, 26, 38, 41]. This exacerbates three ongoing and inter-linked public health pandemics: undernutrition and food insecurity, obesity and diet-related non-communicable diseases (NCDs), and climate change and biodiversity loss – referred to as the “Global Syndemic” (B. A. [32]).

Policymakers working across different and heterogeneous fields (agriculture, fisheries, rural development, health, environment, transport and supply infrastructure, trade, social rights, international cooperation, etc.) make decisions that shape food systems, impacting both population and planetary health through food production and consumption patterns. Sustainable production and consumption can broadly be defined as encompassing any and all issues that seek to improve the way that products and materials are sourced, manufactured, and marketed and the way that products are purchased, used, and disposed of at the end of their useful lives [42]. To achieve global sustainable development, fundamental changes in the way societies produce and consume food are indispensable. In this study, sustainable healthy diets are dietary patterns that promote all dimensions of individuals’ health and wellbeing; have low environmental pressure and impact; are accessible, affordable, safe and equitable; and are culturally acceptable [9]. The aims of Sustainable Healthy Diets are to achieve optimal growth and development of all individuals and support functioning and physical, mental, and social wellbeing at all life stages for present and future generations; contribute to preventing all forms of malnutrition (i.e. undernutrition, micronutrient deficiency, overweight and obesity); reduce the risk of diet-related NCDs; and support the preservation of biodiversity and planetary health [9]. Worldwide, healthy diets are often considered as those dietary patterns rich in health-promoting foods, including plant-based foods, fresh fruits and vegetables, antioxidants, nuts, and sources of omega-3 fatty acids, and low in saturated fats and trans fats, animal-derived proteins, and added/refined sugars [25]. These patterns are commonly part of the cultural and traditional diet in most regions of the world, rooted in local/regional traditions and food sources, as is the case for the traditional Mediterranean and Asian diets [6]. Since the beginning of the century, the world has experienced a wave of globalisation, which has generated lower prices for foods, increased access to a wide variety of foods, and has helped to reduce global poverty [12]. Nevertheless, globalisation has also led to increases in the availability of less healthy foods and ultra-processed foods while exacerbating nutritional and environmental vulnerabilities [2, 16, 31, 36], particularly import volumes of animal products and ultra-processed foods in low- and middle-income countries (LMICs) [1, 10, 17, 18, 20, 34, 35]. The geographical availability of less healthy foods drastically impacts the dietary patterns of lower socio-economic groups [24, 29]. Therefore, the relationship between food systems and global and social inequalities is a controversial topic, as the effects are heterogeneous across countries, settings and households. In addition, the impacts among genders also differ. While food trade and globalisation can improve women’s empowerment by creating new jobs, enhancing food choices and increasing women’s bargaining power in society [28], it can also lead to job losses and a concentration of work in lower-skilled jobs [40].

Hence, the transition in this global context to healthier and more environmentally sustainable food systems is essential to improve our understanding of the effects and effectiveness of public policies on our health and the environment; and even more importantly, to understand their ability to simultaneously reduce the burden of the Global Syndemic, while taking into account potential effects on inequalities and women’s empowerment. For this reason, the double- or triple-duty potential of diverse public policies needs to be identified and evaluated (B. A. [32, 39]).

Ever since the 2021 United Nations Food Systems Summit, there have been growing calls for governments to adopt a holistic “food systems approach” to make progress at policy level that simultaneously tackles these three ongoing pandemics, with coordination to avoid incoherent interventions [8, 13, 14, 23],B. A. [32]. These calls are based on the realisation that one policy could improve multiple outcomes (double- or triple-duty potential), that there could be potential synergies (mutually advantageous effect from the application of one policy on the implementation and/or effectiveness of another policy), but that there can also be trade-offs (negative effects across policy objectives, effectiveness and/or outcomes) when trying to simultaneously reduce food insecurity and undernutrition, obesity and diet-related NCDs, and improving environmental sustainability.

To create healthier and more environmentally sustainable food systems, it is therefore crucial to identify evidence-informed policy options for governments (hereafter referred to as “proposed policies”) that can be used in different contexts and countries globally. In 2013, the International Network for Food and Obesity/NCDs Research, Monitoring and Action Support (INFORMAS) developed the “Healthy Food Environment Policy Index” (Food-EPI) (B. [33]), a tool and process used to assess and benchmark national governments’ actions to create healthy food environments that prevent obesity and NCDs and identify key priority actions for future implementation [37]. In 2018, a team of international scientists created the Food Systems Dashboard [8] a tool used to describe global, regional and national food systems; to assess the challenges for improving diets, nutrition and health, and to guide its users to set priorities and decide on actions. However, to the best of our knowledge, a policy-focused tool that proposes a comprehensive list of double- and triple-duty actions for governments to create healthier and more environmentally sustainable food systems has not been developed yet.

Prior to undertaking this research, we conducted a scoping review to identify the double- and triple-duty potential of different food systems policies that have already been implemented and evaluated. The findings from this scoping review highlighted that some food systems policies, once implemented by governments, have beneficial effects in multiple outcomes analysed (double- or triple-duty potential) [4]. However, not all the proposed policies have been designed or implemented to date, and not all the implemented policies have been evaluated, displaying some important gaps in the evidence available. The key results from this scoping review showed that some of these policies positively impact undernutrition, obesity, and climate change (the three primary outcomes studied). The identified triple-duty policies were (a) sustainable agriculture practices (i.e. agroecology, carbon sequestration, crop rotations, school gardens) and (b) school food programmes. The identified double-duty policies were (a) front-of-pack labelling, (b) in-store nudging interventions, (c) food provision in public sector settings, and (d) fiscal measures (i.e. taxes and subsidies). The scoping review [4] identified one synergy (i.e. a combination of food prices and food retail policies increase healthier purchases) and four trade-offs (i.e. water desalination strategies negatively impact climate change; food provision policies may increase food waste; food labelling may increase nutrition-related inequalities; food subsidies may increase food purchasing with the money saved). More detailed information on the complete methodology applied and the specific results from the scoping review can be found in the respective paper [4].

In this vein, our study aimed to identify and prioritise a comprehensive list of policies for governments to shift populations towards sustainable healthy diets in a way that explicitly considers and integrates the linked outcomes of undernutrition and food security, obesity/NCDs and environmental degradation. In this way, the study informs policymakers on public policies that can be designed, implemented and evaluated at national, regional and/or local levels of jurisdiction to create healthier and more environmentally sustainable food systems.

## Methods

This study aimed to create a list of proposed policies towards healthier and more environmentally sustainable food systems, applicable to governments globally at any desired level of jurisdiction. We started by compiling existing international policy recommendations addressed at governments to identify potential food systems policies, and based on that we conducted a scoping review [4] to examine the effects and effectiveness of those internationally recommended policies on a total of five outcomes. The three primary outcomes were (i) undernutrition, (ii) obesity/NCDs, (iii) environmental sustainability. In addition, (iv) inequalities and (v) women’s empowerment were included as secondary outcomes, as they are not direct outcomes of the Global Syndemic and not considered when assessing the double- or triple-duty potential of policies, but in a non-linear way they are simultaneously drivers and outputs common for the three pandemics. The aspects considered for each outcome are available in Table 1.

**Table 1:** Inclusion criteria of the primary and secondary outcome areas analysed in this study

Outcome areas				
**Primary**			**Secondary**	
**Undernutrition**	**Obesity/NCDs**	**Environmental sustainability**	**Inequalities**	**Women’s Empowerment**
Food insecurity Wasting Stunting Underweight Micronutrient deficiencies (vitamin and/or minerals)	Overweight Obesity Diet-related NCDs (i.e. cancer, cardiovascular diseases, fatty-liver disease, diabetes)	Vegetation and biodiversity loss GHGE Soil erosion Eutrophication Land degradation and desertification Deforestation Acidification Soil, water, and air pollution Water scarcity	Socio-economic inequalities in food access/nutrition Gender inequalities in food access/nutrition	Refers to the process of increasing women’s access to control over the strategic life choices that affect them and access to the opportunities that allow them fully to realize their capacities [7]

*NCDs* non-communicable diseases, *GHGE* greenhouse gas emissions. Chen, YZ., Tanaka, H. (2014). Women’s Empowerment. In: Michalos, A.C. (eds) Encyclopedia of Quality of Life and Well-Being Research. Springer, Dordrecht. https://doi-org.ezproxy.ulb.ac.be/10.1007/978-94-007-0753-5_325

**Fig. 1:**
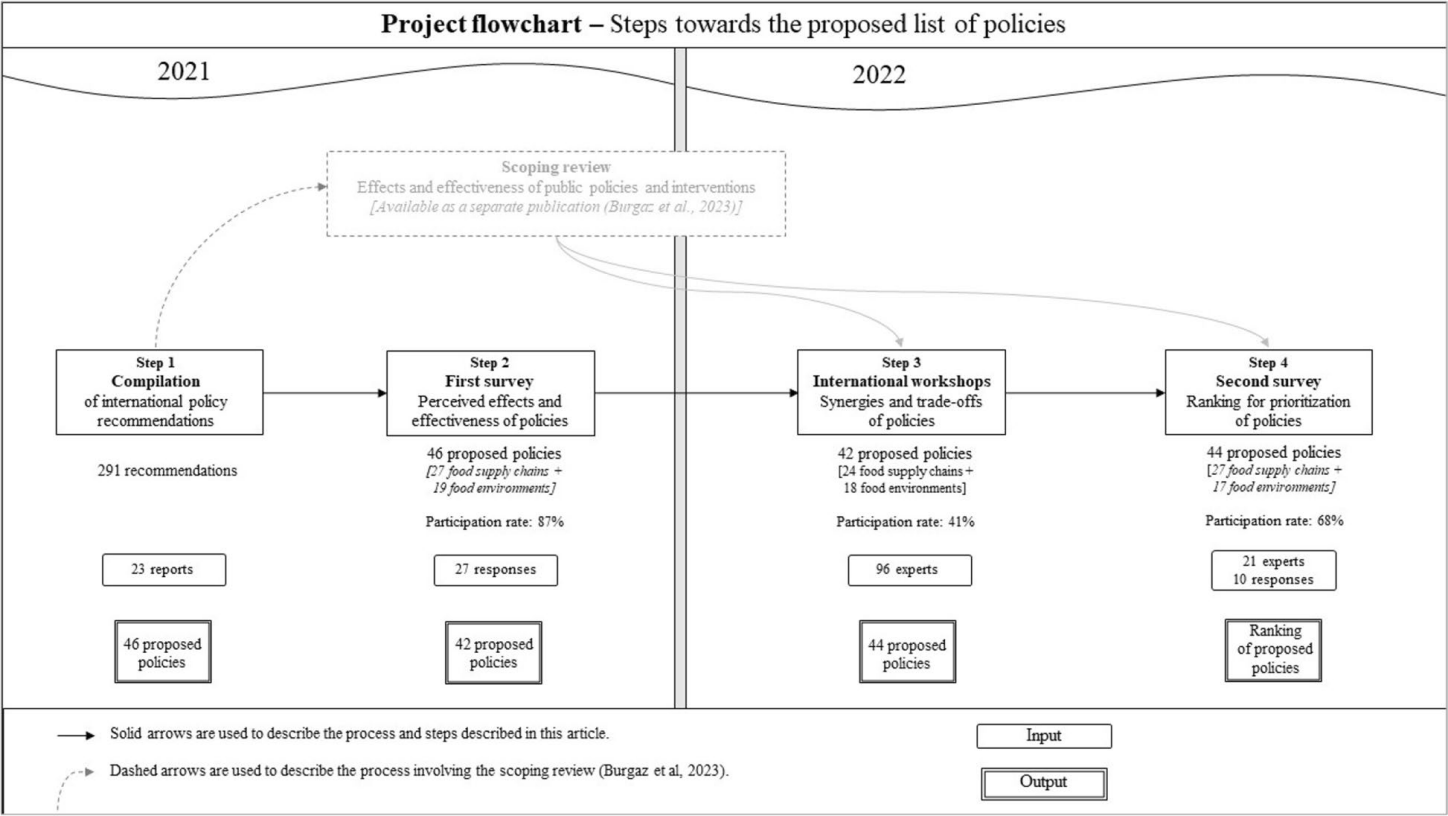
Flowchart of the steps undertaken in 2021–2022 to develop the proposed policies for governments towards healthier and more environmentally sustainable food systems, number of policies considered across each step, and input sources

Due to the lack of evidence available for effectiveness of policies within some policy subdomains on different outcomes [4], additional insights on the list of proposed policies were gathered through an international expert consultation. This was done by conducting two online surveys and four regional workshops to identify the perceived effects (double- or triple-duty potential), the effectiveness, synergies and trade-offs of the list of proposed policies, regardless of their implementation level. All the inputs, changes in the number of proposed policies according to each step, and the complete process are depicted in Fig. 1.

### Step 1 – Compilation of international recommendations

From March to July 2021, we conducted a desk review of international guidelines, reports and peer-reviewed scientific articles that recommended policy actions for governments to improve food systems concerning population nutrition, nutrition-related inequalities and/or environmental sustainability.

The list included documents found through a grey literature search conducted on the Internet and key documents already known to the co-authors involved in this research, mainly consisting on reports from international organisations [namely the Food and Agriculture Organization of the United Nations (FAO), the World Health Organization (WHO), the Global Alliance for Improved Nutrition (GAIN), the Organisation for Economic Co-operation and Development (OECD), the United Nations Children's Fund (UNICEF) and the International Food Policy Research Institute (IFPRI)]. To be eligible for inclusion, each report/guideline/paper was assessed against the three following criteria: (i) it contains specific, detailed recommendations for government policies or actions addressing food systems, and policy recommendations needed to be action-oriented and specific (ii) it contains information details about the funding sources; reports/guidelines produced by the agriculture or the food industry were not included (iii) it was published between 2011 and 2021. A total of 23 documents met these inclusion criteria and were used as sources for the compilation. The 23 documents were reviewed in detail, extracting a total of 291 policy recommendations for governments (Annex 1).

As foreseeable, overlaps were found across the policy recommendations. In such cases, they were merged keeping the content of the original recommendations as close as possible to how they were worded. This allowed both for the identification of potential policy areas to be addressed (that in this research I refer to as ‘domains’ and ‘subdomains’) and the generation of a shorter, merged list of policies for governments (Table 2).

**Table 2:** Recommendations extracted and merged, divided by policy domains and subdomains

Policy domains	Policy subdomains	Recommendations	Merged
**Food supply chains**	Food production	86	15
	Food storage, processing, packaging and distribution	16	6
	Food loss and waste	20	3
	Food trade and investment	19	3
**Food environments**	Food composition	12	2
	Food labelling	18	4
	Food promotion	31	2
	Food provision	23	3
	Food retail	26	4
	Food prices	40	4
		**291**	**46**

All the recommendations were compiled and classified according to the food systems areas they were addressing in these ten subdomains, that were identified in two ways: for the food supply chains domain, an inductive approach was used based on the thematic areas of the policy recommendations; for the food environments domain, a deductive approach based on the Food-EPI tool was adopted (B. [33]). Once classified and merged per domain and subdomain, 46 proposed policies were retained, as they covered individual food systems aspects and proposed policy actions that could tackle one of more of the three outcomes studied. To ensure consistency across the policies recommended, the same approach and languages used for the Food-EPI were adopted in the phrasing of the recommended policies (B. [33]). The results of this compilation were used both to create the first survey to identify the perceived effect and effectiveness of the proposed policies (step 2).

### Step 2 – First survey: perceived effects and effectiveness of the recommended policies

In November 2021, an online survey (LimeSurvey) was conducted among international agriculture, food and environmental sustainability experts. The objective of the survey was to get insights from experts on the perceived double- and triple-duty potential of the proposed policies, and to identify potential synergies and trade-offs across the outcomes (Table 1).

Experts were recruited within two networks: (1) the INFORMAS network and (2) the Food Sustainability Advisory Team (Food-SAT), which was established under the INFORMAS2.0 IDRC project.^1^

Two surveys were created, one for the domain of food supply chains and one for food environments. Experts were asked to assess the potential effect of 46 recommended policies on the five outcomes. Experts could rate the effect of the policies as “positive”, “negative”, “neutral”, “non-applicable” or “unknown”. When experts selected “positive” as the perceived effect, they were asked to rate the effectiveness according to three levels: “very effective”, “effective” or “somewhat effective”. Therefore, each expert had to assess the effect and effectiveness level of each policy across the outcomes, to allow for the identification of policies with double- or triple-duty potential. At the end of the survey, we asked for additional feedback or suggestions for us to take into account during the analysis of the results or the further development of the list of proposed policies.

The survey responses were analysed per policy, considering their double- or triple-duty potential, and the potential synergies or trade-offs across the three primary outcomes. Based on the results of the survey, the policies were classified according to three categories: (1) essential to keep (those perceived as likely to have a strong positive impact on at least two outcomes, with no negative impacts perceived); (2) to be excluded (those considered likely to have a negative impact on at least one primary outcomes, with only low effectiveness in all others); and (3) policies with mixed results (those with perceived mixed effects, either likely to have strong effectiveness in two or three primary outcomes with negative impacts in others, or those identified as important single-duty policies which were likely to have strong effectiveness in just one outcome but without perceived effects in the others). The results of this survey were used to inform the experts participating in the regional workshops and to advise on the final selection of the policies.

### Step 3 – Regional workshops

Between May and July 2022, four online workshops were organised. We invited agriculture, food, health and environmental sustainability experts from the regions included in the INFORMAS2.0 project (Europe, Latin America, East and West Africa). The objective of each of the workshops was to discuss the perceived effect (double- or triple-duty potential) of the proposed policies on the five outcomes. Based on their organisation, role, and relevant research field, experts from the four regions were identified and contacted by the INFORMAS2.0 partners in Belgium, Brazil, Kenya and Senegal. In order to be included, experts were assessed according to two main criteria: (i) he/she is directly involved in at least one of the ten food systems subdomains identified (either conducting research or through the design or implementation of policies); (ii) his/her country of origin belongs to the four regions analysed. A total of 235 experts were invited via email, with the request to reply if they were interested in participating. Their written confirmation was used as their consent. Prior to the workshop, the participating experts were divided into four different sub-groups (based on their field of expertise) to ensure optimal feedback within all subdomains. The distribution of the 96 experts who participated in the workshops, their field of expertise, type of organisation, country of origin and their assigned groups can be found in Annex 2.

The workshops were organised in English (for Europe and East Africa), Spanish (for Latin America) and French (for West Africa). A short introduction was given at the beginning of the workshop, after which participants were divided into breakout rooms according to their assigned sub-group and were invited to reflect on the following aspects of each proposed policy: (i) the content clarity and wording; (ii) the level of (dis)aggregation; (iii) the double-/triple-duty potential and its potential effect on inequalities and women’s empowerment; and (iv) any potential synergy or trade-off across the outcomes. The information on the policies’ double- or triple-duty potential, as well as the synergies/trade-offs identified through the scoping review [4] and the first survey was provided for each proposed policy. Experts were also asked to suggest additional double- or triple-duty policy options that were not covered in the proposed list.

The final list of proposed policies was created taking into account the findings from the scoping review, the survey and the feedback from the workshops. The results gathered through the scoping review were prioritised. When scientific evidence was not available, the input from the experts was considered. During the process of combining the results from the different steps, some policies were regrouped, others were disaggregated and others were reworded. Suggestions made by experts that were out of scope [as they did not directly impact the five outcomes, but had a more upstream focus (i.e. other social or economic determinants)] were not taken into account. All trade-offs identified were considered when creating the list of proposed policies, and for some policies experts identified potential solutions and proposed changes in the text to reduce or eliminate their negative impact. However, given the complexity of food systems and the differences across countries and contexts, it was not always possible to modify the policy wording to address all the potential trade-offs identified during the discussions. For those cases, the trade-offs were simply noted down.

### Step 4 – Second survey: prioritisation

In October 2022, an additional meeting with the Food-SAT (*n* = 21) and INFORMAS2.0 (*n* = 10) experts was organised. In this last step, we sought to further verify the proposed changes and to identify which policies they considered should be prioritised. The 31 experts were invited via email, and to enable maximum participation, two meetings at different times/dates were organised. Before the meeting, the new list of proposed policies (resulting from step 3), the results from the prior conducted scoping review [4] and the feedback received from the workshops (step 3) were shared with them. During the meetings, experts were asked to share their feedback on the list of proposed policies, based on the scientific evidence available and their expertise in the field.

After the session, experts were asked to rank the proposed policies according to their perceived effectiveness to improve one (or more) primary and/or secondary outcome(s) of their choice. In order to rank them, experts had to select from the list of proposed policies the ones that had a positive effect on the chosen outcome(s). Once selected, experts had to order them according to their (perceived) level of effectiveness. Given the difficulty of comparing the importance of policies across the two core domains, the ranking was done separately for the 27 food supply chains policies and the 17 food environments ones. There was no limit to the number of policies that could be ranked. However, in order to ensure that a significant ranking among policies was conducted, experts had to select a minimum of 5 per domain. At the bottom of the survey, experts could give additional explanations or comments to be taken into consideration while analysing the data.

A numerical value was assigned to the ranking positions: the policy in the highest position in the ranking was assigned a value of 10, the second policy an 8, the third a 6, the fourth a 4, the policy in the fifth position a 2, and all the other policies that were selected and ranked in lower positions (below the fifth) were given a value of 1. This was done to ensure a distinction between policies that were considered relevant but in lower positions in the ranking, versus those that were not selected at all (therefore perceived as not effective for the chosen outcome). This way, we were able to identify the proposed policies considered to be prioritised, by domain and by outcome. The extent to which experts agreed on the level of priority of the proposed policies was then analysed by policy domain and outcome in Excel using Gwet AC2 inter-rater reliability coefficient with Agreestat360.

## Results

### List of proposed policies for governments

Using a participatory and transdisciplinary approach involving international experts to identify the effects, effectiveness, and potential dynamics that lead to synergies and trade-offs across outcomes, we propose a list of 44 policies for governments to create healthier and more environmentally sustainable food systems from SFS: 27 for food supply chains and 17 for food environments. The complete list is available in Table 3. In this list, there is no hierarchy to how the proposed policies are presented, as they are classified according to the prior defined domains and subdomains (Table 1).

**Table 3:** List of the 44 proposed policies for creating sustainable food systems that have the potential to address undernutrition, obesity and environmental sustainability simultaneously, noting potential synergies and trade-offs

**Domain: Subdomain** **Title** Good practice statement	**Primary outcome areas**			**Secondary outcome areas**		**Synergies and trade-offs** (underlined when addressed in the text)
	Undernutrition	Obesity	Env. Sustainability	Social inequalities	Women’s empowerment	
**Food supply chains: Food production**						
**Sustainable carbon sequestration practices** The government has programmes in place to (non-)financially support agroecological practices that enhance natural carbon capture, such as: ** ◦**Bio-sequestration practices through agroforestry systems (e.g. agrosilvocultural, agrosilvopastoral); ** ◦**Afforestation or reforestation practices; ** ◦**Use of cover crops; ** ◦**Organic farming restoration of agriculturally degraded lands			✔			**-**
**Sustainable fisheries** The government’s national fisheries management policy directives guarantee that fishing activities carried out by the national fleet are environmentally sustainable (including artisanal fish and other aquatic systems) and require fully-documented fisheries taking into account bycatch and the preservation of marine ecosystems and sensitive species				✔		**Trade-off:** Undernutrition – if not designed properly, may increase food insecurity and dependence on imports
**Regenerative agriculture** The government has programmes in place to (non-)financially support regenerative agriculture practices that protect the soil, enhancing its fertility and health through: ** ◦**Sustainable soil remediation methods (e.g. bioremediation); ** ◦**Increase of soil's organic matter content; ** ◦**Nitrogen fixation practices and rotation systems; ** ◦**Crop rotation of the land and return of crop residues		✔	✔			**Synergy:** PRODUCT16 | Climate change impact preparedness – may improve resistance to droughts, severe heat and aberrant precipitations
**Optimisation of water resources management** The government requires farmers to integrate sustainable water resource management practices (e.g. managed aquifer recharge and storage, subsurface waste storage and injection) that optimise water resources and agricultural outputs per unit of water			✔	✔		**-**
**Incentives for crop, fish and livestock diversification** The government provides (non-)financial incentives to farmers and fishers to implement sustainable diversification practices, such as polyculture or crop-livestock integration, in: ** ◦**Food crops (including neglected and underutilised traditional crops); ** ◦**Livestock species; ** ◦**Fisheries (including algae and new/emerging finfish species)	✔	✔	✔	✔		**Trade-off:** Undernutrition – may increase the prices of final food products
**Evidence-based use of bio-fortification programmes** The government has implemented a plan to prevent micronutrient deficiencies among the population, according to a hierarchy scheme in which priority will be given to the promotion of healthier and more sustainable crops that are part of a diversified diet. The plan establishes clear evidence-based criteria according to national guidelines (and food security programmes, if any) to justify the use of nutrition-sensitive agriculture and bio-fortification programmes for healthier and more sustainable crops	✔		✔			**Trade-off:** Undernutrition & environmental sustainability – may increase prices of final food products, or incentivise the use of genetically modified organisms
**Land use management** The government has regulations in place that restrict how the land can be used to support environmental conservation, and address desertification and land degradation, including: ** ◦**Financial incentives to farmers in exchange for providing ecosystem services when managing the land and working on the land; ** ◦**Guidance on land use that encourages sustainable development	✔		✔	✔	✔	**-**
**Evidence-based reduction of the use of fertilisers** The government has regulations in place to reduce, based on scientific evidence*, the synthetic use of fertilisers, including: ** ◦**Targets for reductions of nitrogen and phosphorus discharges; ** ◦**Fertilisers accounting systems; ** ◦**Nitrogen quota systems; ** ◦**Taxes on phosphorus content in feed; ** ◦**Promotion of micro-dosing techniques; ** ◦**Support for the use of natural fertilisers (such as manure or compost) in large areas of land; ** ◦**Protection of groundwater and coastal areas from fertiliser pollution		✔	✔			**Trade-off:** Undernutrition – if not designed properly, may reduce productivity and increase food insecurity and dependence on imports
**Evidence-based reduction of the use of pesticides** The government has regulations in place to reduce, based on scientific evidence, the use of chemical pesticides, including: ** ◦**Avoiding the use of hazardous chemical pesticides; ** ◦**Promoting best practices to minimise the associated risks to human health and the environment; ** ◦**Encouraging farmers to use sustainable prevention techniques for disease and pest management		✔	✔			**Trade-off:** Undernutrition – if not designed properly, may reduce productivity and increase food insecurity and dependence on imports
**Subsidies for sustainable healthy crops/livestock/fish** Existing subsidies by the government intend to support sustainably produced crops, livestock and fish that contribute to healthier and more sustainable diets. These subsidised crops, livestock and fish shall be for food products intended for human consumption, and not for animal feed	✔	✔	✔	✔		**-**
**Farmers’ access to traditional seeds and breeds** The government has programmes in place that protect ancestral, traditional seeds and breeds, such as: ** ◦**Making them economically accessible to smallholder/family farmers, prioritising them over industrial varieties; ** ◦**Declaring ancestral seeds and breeds as public goods (property of the local community)		✔	✔	✔	✔	**-**
**Farmers and fishers' support** The government has programmes in place to support farmers and fishers taking part in the transition towards sustainable food systems, such as: 1. Subsidizing access to sustainable technologies and innovation (including digital technologies); 2.Providing skill training and capacity development programmes on sustainable and regenerative practices, and climate change adaptation		✔	✔	✔	✔	**Trade-off:** Environmental sustainability – if not designed properly, the innovation and technologies may be harmful if they incfertilisersizers, genetically modified organisms, antibiotics and pesticides
**Support for women's Empowerment** The government has programmes in place that enhance the participation of women and give (non-)financial support to women in agriculture and fisheries, such as: ** ◦**Providing start-up grants, loans or guarantees (designed to help or advice on how best to enter sustainable farming); ** ◦**Guaranteeing decision-making power in leadership positions; ** ◦**Providing income support for sustainable technologies and sustainable innovation; ** ◦**Providing subsidies for land/infrastructure and storage facilities; ** ◦**Providing capacity development programmes for female farmers and fishers on sustainable and regenerative practices, and climate change adaptation; ** ◦**Prioritize female farmers and fishers when it comes to receiving basic payment entitlements from the national/regional reserve; ** ◦**Prioritize female farmers and fishers’ access to local, national and international markets; ** ◦**Recognising and securing legitimate tenure right holders and their rights with customary tenure systems that exercise self-governance of land, forests, fisheries and/or water Equitable opportunities to earn and learn shall be compatible with safe pregnancy and breastfeeding	✔	✔	✔	✔	✔	**-**
**Support to young generations** The government has programmes in place that give (non-)financial support to young generations (< 40) in sustainable agriculture and fisheries, such as: ** ◦**Providing start-up grants, loans or guarantees (designed to help or advice on how best to enter sustainable farming); ** ◦**Guaranteeing decision-making power in leadership positions; ** ◦**Providing income support for sustainable technologies and sustainable innovation; ** ◦**Providing subsidies for land/infrastructure and storage facilities; ** ◦**Providing capacity development programmes for young farmers and fishers on sustainable and regenerative practices, and climate change adaptation; ** ◦**Prioritize young farmers and fishers when it comes to receiving basic payment entitlements from the national/regional reserve; ** ◦**Prioritize young farmers and fishers’ access to local, national and international markets; ** ◦**Recognising and securing legitimate tenure right holders and their rights with customary tenure systems that exercise self-governance of land, forests, fisheries and/or water		✔	✔	✔		**-**
**Ecosystem restoration and conservation** The government has programmes in place to conserve and protect biodiversity across: ** ◦**Lands and forests (zero-expansion policy of new agricultural land, restoring and reforesting degraded land) ** ◦**Waters, oceans and coastal seas (marine protected areas, mangrove forests) Adopting a "Half-Earth" strategy for biodiversity conservation	✔		✔	✔		**Trade-off:** Undernutrition – if not designed properly, may be negative for indigenous communities owning the land
**Climate change impacts preparedness** The government has programmes in place for climate change impact preparedness that enhance the resilience of food supply chains, such as: ** ◦**weather insurance for farmers and fishers; ** ◦**interventions to reduce and manage price volatility; in case of pests, diseases, weather-related shocks or emergencies			✔	✔		**-**
**Food supply chains: Food storage, processing, packaging and distribution**						
**Connecting smallholder farmers with territorial markets** The government has programmes in place to guarantee that the local, fresh and diverse production of smallholder/family farmers reaches territorial markets (urban, peri-urban and rural), such as: ** ◦**Investing in railway (preferred) or road transportation and infrastructure; ** ◦**Investing in storage facilities to be rented at lower prices to smallholder/family farmers; ** ◦**Providing grants allocated to the construction of storage facilities in-farm; ** ◦**Covering the costs associated with hygienic practices for food storage and handling before selling; ** ◦**Tax exemptions on infrastructure use Priority is given to smallholder/family farmers coupled with organic farming, agroecology or sustainable agriculture practices	✔	✔	✔	✔		**Trade-off:** Environmental sustainability – if the infrastructure supported increases, the use of roads or aeroplanes will be harmful to the environment. Train infrastructure should be prioritised
**Support for start-ups and small and medium-sized enterprises producing more sustainable and healthier foods** The government provides (and if already in place, makes good use of) investment funds for start-ups, and small- and medium-sized food businesses that store, process, package and/or handle healthier and more sustainable foods. A fixed amount of the funding is addressed to employment generation in rural areas and distant locations	✔	✔	✔			**-**
**Evidence-based use of fortification programmes** The government has implemented a plan to prevent micronutrient deficiencies among the population, according to a hierarchy scheme in which priority will be given to the promotion of healthier and more sustainable foods as part of a diversified diet. The plan establishes clear evidence-based criteria according to national guidelines (and food security programmes, if any) to justify the use of either: ** ◦**Mandatory large-scale food fortification programmes to increase the availability of nutrients (e.g. iron, folate, zinc) in healthier and more sustainable foods available to all population groups; ** ◦**The distribution of healthier and more sustainable micronutrient-fortified foods targeted at specific population groups; ** ◦**Supplements targeted at population groups severely affected	✔	✔				**Trade-off:** Obesity/NCDs – Some fortified products are unhealthy, ultra-processed foods
**Environmental impact measures** The government requires the food-producing companies to measure the environmental impact of individual food and drink products by calculating in a standardised way an eco-score (e.g. life-cycle analysis, full environmental footprint) for the environmental impact		✔	✔			**Trade-offs:** All outcomes – (a) May increase the final product price. (b) It would only be applicable for processed foods, not fresh ones (c) The environmental impact assessment will never be perfect, given the numerous variables to consider (with different hierarchies according to the context)
**Food supply chains: Food loss and waste**						
**Food loss prevention and reduction through infrastructure investment** The government minimises food losses across the whole food supply chain by investing in sustainable infrastructure that improves post-harvest techniques and fosters the development of facilities for post-harvest storage, food processing and transportation	✔		✔			-
**Food loss and waste reduction through a step-wise process** The government requires all stakeholders across the food supply chain to have programmes in place that reduce (and ideally eliminate) food loss and waste, such as: ** ◦**Elaborating a prevention plan according to a hierarchy of uses for food fit for consumption but consciously discarded, in which priority will be given to human consumption: ** ◦**Priority: fiscal measures, such as tax breaks or price adjustments, giving supremacy to fresh, healthier and more sustainable foods; ** ◦**Second step: human consumption through a donation to organisations (such as food banks), giving supremacy to fresh, healthier and more sustainable foods, and avoiding (if possible) ultra-processed foods. Companies should sign agreements with the receiving organisations specifying the conditions for collection, storage and transport ** ◦**Third step: to transform food into other edible products for human consumption ** ◦**Fourth step: to use the food as animal feed ** ◦**Last uses: processing of industrial by-products and recycling into compost or fuels ** ◦**Implementing a mandatory food waste reporting system The government contemplates financial penalties for institutions that do not comply with this legislation	✔	✔	✔	✔		**Trade-off:** Undernutrition – Incentivizing donations may maintain a system of poor quality and low diversity production surpluses reused in the food aid system, often financed by public expenditure. Such spending could support sustainable food options (small-scale, agroecological and local)
**Regulation framework at the retail level** The government has programmes in place to minimise food waste at the retail level: ** ◦**Bars, cafeterias and restaurants shall be obliged to make it possible for consumers to take home food that they have not consumed, at no additional cost. To this end, reusable or easily recyclable packaging suitable for food use must be available to the consumer; ** ◦**Supermarkets shall have sales lines for "ugly, imperfect or un-aesthetic" products, promoting the consumption of seasonal, healthier and/or more sustainable products		✔	✔			**Trade-off:** Obesity/NCDs – May only be applied to processed foods with expiration dates, not to fresh products
**Food supply chains: Food trade and investment agreements**						
**Risk impact assessment of trade and investment agreements** The government conducts cross-ministry and cross-sector risk impact assessments before the negotiation and implementation of multilateral and bilateral trade and investment agreements, to identify, evaluate and minimise the direct and indirect impacts of such agreements on the population's nutrition and health, food security and environmental sustainability, both domestically and in third countries. The assessment includes an analysis of trade-offs interconnected to these factors. Focus is given to reducing health and gender inequalities	✔	✔	✔	✔		**Trade-off:** All outcomes – Trade liberalisation is a very political topic, always linked to trade-offs, very context-specific (effective trade tools may vary)
**Effective use of trade policy levers for sustainable food systems** The government adopts measures to manage investment and protect its regulatory capacity from the influence of vested interests on policy and uses trade policy levers more effectively to ensure environmental sustainability and the availability and accessibility of healthier and more sustainable foods, prevent overweight and obesity, reduce food insecurity and under-nutrition, and promote food policy space. Instruments such as appropriate tariffs can help shift foods available domestically as well as their relative prices	✔	✔	✔	✔		**Trade-off:** All outcomes – Trade liberalisation is a very political topic, always linked to trade-offs, very context-specific (effective trade tools may vary)
**Trade incentives for shorter food supply chains** The government provides trade policy incentives that enable shorter food supply chains linking cities to secure a supply of healthier and more sustainable foods and ensuring that priority is given to food from exports and imports that do not contribute to deforestation, land use change, biodiversity loss, eutrophication, or to the displacement of vulnerable groups from their traditional lands Priority is given to shorter food supply chains coupled with organic farming, agroecology or sustainable agriculture practices		✔	✔	✔		**-**
**Transparency of global food supply chains** The government fosters sustainable and responsible corporate behaviour throughout the global value chains, such as: ** ◦**Implementing Participatory Guarantee System (PGS) for sustainable production; ** ◦**Requiring companies to identify and, where necessary, prevent, end or mitigate adverse impacts of their activities on the environment (e.g. pollution, use of hazardous chemicals, biodiversity loss)		✔	✔	✔		**Trade-off:** Undernutrition – may increase the prices of final food products, as standards and certifications are often expensive and bureaucratic processes
**Food environments: Food composition**						
**Reformulation of processed foods** Food composition targets/ standards/ restrictions/ bans have been established by the government for: ** ◦**the content of energy, nutrients of concern (trans fats, added sugars, saturated fat) and sodium; ** ◦**the use of less healthy and less sustainable ingredients (e.g. palm oil, animal protein sources); ** ◦**the portion sizes of processed foods		✔	✔			**Trade-offs:** Undernutrition and obesity/NCDs – (a) may increase prices of final food products. (b) even if foods are reformulated to fit standards, new formulations used may not necessarily be healthier
**Reformulation of out-of-home meals** Food composition targets/ standards/ restrictions/ bans have been established by the government for: ** ◦**the content of energy, nutrients of concern (trans fats, added sugars, saturated fat) and sodium; ** ◦**the use of less healthy and less sustainable ingredients (e.g. palm oil, animal protein sources); ** ◦**the portion sizes of meals sold by food service outlets, food delivery services and informal food outlets (e.g. food street vendors)		✔	✔			**Trade-off:** Obesity/NCDs – even if foods are reformulated to fit standards, new formulations used may not necessarily be healthier
**Food environments: Food labelling**						
**Nutrition information panels and ingredient lists** ** ◦**Ingredient lists and nutrient declarations in line with the most recent Codex recommendations are present on the labels of all packaged foods ** ◦**Regulations ensure that information concerning the quantity of added sugar and trans-fat within a food product is presented clearly in the nutrition information panel of all relevant packaged foods ** ◦**Regulations ensure that information concerning the type of all fats and oils (e.g. palm oil, sunflower oil) and added sweeteners (e.g. fructose, dates, honey) within a food product is presented in the ingredient list of all relevant packaged foods		✔	✔			-
**Evidence-based claim regulations** Robust, evidence-based regulatory systems are in place for: ** ◦**Approving/reviewing nutrition and health claims on foods, according to an evidence-based independent nutrient profile model; ** ◦**Approving/reviewing environmental sustainability claims so that consumers are protected against misleading claims on packaged foods which lack scientific evidence		✔	✔	✔		**Trade-offs:** All outcomes – (a) may increase prices of final food products. (b) may deviate attention and reduce the impact of other interventions, such as other labels (Front-of-pack labelling)
**Front-of-pack nutrition/environmental labelling** A mandatory, consistent, evidence-informed front-of-pack label with a: ** ◦**Nutrition information system (e.g. warning labels, numerical/colour-coded, graded indicators) ** ◦**Environmental information system (e.g. lifecycle analysis-inspired label, full environmental footprint label) is required for all packaged foods (either combined or separate), which readily allows consumers to assess a product's healthiness and/or environmental impact		✔	✔			**Synergy:** COMP1 | Reformulation of processed foods – incentivises the reformulation of food products by the industry **Trade-offs:** Undernutrition – may increase prices of final food products
**Out-of-home eating outlets’ menu labelling** A consistent, simple, clearly-visible system of menu labelling is required for all out-of-home eating outlets (including vending machines) with a: 1.Nutrition information system (e.g. energy content, the content of nutrients of concern, warning labels, numerical/colour coded, graded indicators); 2.Environmental information system (e.g. lifecycle analysis-inspired label, full environmental footprint label); for all foods and meals on sale		✔	✔	✔		**-**
**Food environments: Food promotion**						
**Marketing restrictions of less healthy and less sustainable foods to children across all media** Comprehensive, mandatory regulations with effective enforcement are implemented by the government to restrict exposure and power of promotion of less healthy and less sustainable foods to children, and/or their parents/caregivers across all types of media, including: 1.Broadcast media (television, radio); 2.Non-broadcast media (e.g. internet, online videogames, influencers/social media); 3.Food packaging; 4.Sports clubs and events (e.g. sponsorship, billboards/advertising posters); 5.In public settings where children gather (e.g. outdoor advertising around preschools, schools, train stations, buses and cultural events)	✔	✔	✔	✔		**-**
**Marketing restrictions on less healthy and less sustainable food in retail outlets** The government has regulations in place to restrict the marketing of less healthy, less sustainable foods within supermarkets and other retail stores, including restrictions on product placement in prominent in-store positions (e.g. checkouts, end-of-aisle displays), price discounts, and sales targeting children and/or their parents/caregivers		✔	✔	✔		**-**
**Marketing restrictions on breastmilk substitutes** The government implements, across all media and settings, the policies and practices of the 'International Code of Marketing of Breast-milk Substitutes' and 'World Health Assembly Resolutions' (e.g. prohibit the promotion of breastmilk substitutes, feeding bottles and teats) and restricts the marketing of breastmilk substitutes to adult women of reproductive age, pregnant women, parents of children under three years, caregivers and healthcare providers	✔	✔	✔			**Trade-off:** Women’s empowerment – may be negative for women who need to work
**Food environments: Food provision**						
**School food and nutrition policies** The government implements clear, consistent policies in schools and early childhood education services with food service activities (nutrition standards for canteens, food at events, fundraising, promotions, vending machines etc.) that: 1.Promote safe drinking water (safe tap water or water fountains); 2.Provide and promote healthier and more sustainable foods based on nutritional guidelines; 3.Prioritize the purchase of seasonal, healthier and more sustainable foods; 4.Restrict less healthy and less sustainable foods and beverages	✔	✔	✔	✔		**Synergy:** COMP1 | Reformulation of processed foods – incentivises the reformulation of food products by the industry **Trade-off:** Environmental sustainability – if not designed properly, increases food waste
**Public sector setting (other than school) food and nutrition policies** The government has clear, consistent policies in place to guarantee that public procurement and public sector settings (other than school and early childhood education services) with food service activities (e.g. hospitals and healthcare facilities, universities, nutrition standards for public canteens, public events, food banks, armed forces, prisons): 1. Promote safe drinking water (safe tap water or water fountains); 2. Provide and promote healthier and more sustainable food choices based on nutritional guidelines and prioritising the purchase of seasonal, nutritious and sustainable foods; 3. Restrict less healthy and less sustainable foods and beverages	✔	✔	✔	✔		**Synergy:** COMP1 | Reformulation of processed foods – incentivises the reformulation of food products by the industry **Trade-off:** Environmental sustainability – if not designed properly, increases food waste
**Accessibility of shorter food supply chains to consumers** The government gives financial support to shorter food supply chains initiatives that connect farmers and consumers, such as: 1. Community-Supported Agriculture; 2. Promoting the direct sale of agricultural products by farmers on territorial markets without intermediaries; 3. On-farm sales or off-farm schemes (farmers markets, delivery schemes); 4. Producer-to-consumer networks; 5. Collective sales towards public institutions; 6. Other policy tools, such as training and knowledge exchange in marketing and communication on shorter food supply chains, shall (or, if already in place, continue) to be funded by Rural Development programmes Special focus is given to smallholder/family farmers coupled with organic farming, agroecology or sustainable agriculture practices		✔	✔	✔		**-**
**Food environments: Food retail**						
**Zoning laws for healthier, more sustainable retail outlets** 1. Zoning schemes, regulations and policies (e.g. tax incentives) are robust enough and are being used, where needed, by the government to place limits on the density or placement of quick-service restaurants or other outlets selling mainly less healthy and less sustainable foods 2. Zoning schemes are in place to attract healthier and more sustainable grocery stores, kiosks or markets with a focus on underserved, low-income areas		✔	✔			**-**
**Prominence of healthier, more sustainable foods in the (in)formal food sector** The government requires prioritisation of the placement and prominence of healthier and more sustainable foods and the limitation of the in-store availability of less healthy and less sustainable foods, for: 1. Formal food vendors (including food stores and supermarkets); 2. Informal food vendors (including food trucks and street vendors)	✔	✔	✔	✔		**-**
**Food environments: Food prices**						
**Taxes on less healthy, less sustainable foods** 1. The government imposes taxes on less healthy foods (e.g. foods high in nutrients of concern or nutrients of concern in foods) to increase the prices of these foods by at least 20% to discourage less healthy food choices where possible 2. The government imposes taxes on less sustainable foods and/or ingredients (e.g. red and processed meat, palm oil) to increase the prices of these foods by at least 20% to discourage less sustainable food choices where possible 3. These tax revenues are ideally re-invested to create healthier and more sustainable food systems, re-invest in infrastructure (e.g. quality of tap water) or value-added tax reductions of healthier and sustainable foods or beverages		✔	✔	✔		**Trade-off:** Undernutrition & environmental sustainability – (a) taxes are context-specific, and may increase micronutrient deficiencies across vulnerable groups. (b) a tax on sugar-sweetened beverages may increase bottled water purchases and maintain packaging pollution
**Subsidies for healthier and more sustainable foods** The intent of existing subsidies/or value-added tax reductions by the government is for healthier and more sustainable foods (e.g. fruits, vegetables, beans and lentils, whole grains)	✔	✔	✔	✔		**-**
**Affordability of healthier and more sustainable diets** The government requires existing food-related income support programmes (e.g. vouchers, cash, school feeding) to make healthier and more sustainable foods accessible for low-income households and vulnerable groups	✔	✔	✔	✔		**Trade-offs:** Obesity/NCDs & environmental sustainability – (a) saved money from vouchers or cash can be used to purchase unhealthier products. (b) may increase food waste

*NCDs* Non-communicable diseases

^*^The complete reduction of fertilisers can only be made in large areas of land and for "long-term crops" (such as tea or coconut), but not for "short-term crops" (such as rice)

The wording, (dis)aggregation level, classification, and number of policies changed across the process, based on the inputs and outputs of each step of the process (Fig. 1).

The subdomain with more policies was ‘food production’, with 16 proposed policies. The subdomains of ‘food storage, processing, packaging and distribution’, ‘food trade and investment agreements’ and ‘food labelling’ included 4 proposed policies each, followed by ‘food loss and waste’, ‘food promotion’, ‘food provision’ and ‘food prices’ with 3 policies each. Two subdomains (‘food composition’ and ‘food retail’) included 2 policies each.

### Perceived double- and triple-duty potential, synergies and trade-offs

The results from the initial survey showed that 61% of the proposed policies (*n* = 28) were considered by respondents to have double- or triple-duty potential. However, after applying the modifications suggested by the experts during the survey analyses and the regional workshops, the final list included 91% of proposed policies (*n* = 40) with perceived double- or triple-duty potential (Table 3). A total of 25 policies were perceived to have double-duty potential (one for ‘undernutrition’ and ‘obesity/NCDs’, four for ‘undernutrition’ and ‘environmental sustainability’, and 20 for ‘obesity/NCDs’ and ‘environmental sustainability’). A total of 15 policies were perceived to have triple-duty potential. The full results on the perceived effects and effectiveness of the 46 initially proposed policies can be found in Annex 3.

During the regional workshops, three potential synergies and thirty-one trade-offs identified. According to the experts, five trade-offs (out of the 30) could be minimised or avoided in some contexts by adding specific requirements in the proposed policies. The five changes proposed by experts are underlined in Table 3. The outcome with more trade-offs identified was ‘undernutrition’ (*n* = 14), mostly related to lower yields or the potential increase in prices of final products as a consequence of the policy. Eleven trade-offs were identified for ‘environmental sustainability’, mainly regarding the increase of greenhouse gas emissions (GHGEs) from transport, packaging, or food waste. A total of seven trade-offs were identified for ‘obesity and NCDs’, mainly regarding the fact that some of the policies (e.g. on waste, reformulation or labelling) would only be implemented in foods with packaging, nutrition facts or expiration dates, which tend to be unhealthier/processed and not for fresh, natural products. Some of the detected trade-offs applied to more than one outcome. All the identified perceived double- or triple-duty potential, synergies and trade-offs are also available in Table 3.

In addition, three proposed policies were removed as experts considered they were only beneficial for one outcome, with low effectiveness levels and potentially negative for other proposed outcomes. These policies were: (1) reduction of plastics in food packaging (perceived to have a low impact on environmental sustainability but potential negative effects for undernutrition), (2) regulations from governments to reduce water use in farming (perceived to have an impact on environmental sustainability but potential negative effects for undernutrition) and (3) awareness campaigns for food waste reduction (perceived to have a low impact on environmental sustainability and no effects in the other outcomes).

### Implementation considerations

During the workshops, experts stressed that the relevance of some proposed policies may be context-specific, such as in the case of: (1) the need to include water or implement water fountains as part of school food and nutrition programmes; (2) the need to reduce meat consumption (which was considered to be less applicable in contexts where current consumption is very low, with high rates of undernutrition, food insecurity and micronutrient deficiencies); (3) the use of labels (among countries that have implemented warning labels, countries with healthy score labels, and countries without any label and voluntary ingredient list agreements); (4) strategies related to food loss (experts in Europe did not consider policies in this area a priority as the rates in food losses during harvest and transport are often very low); (5) strategies related to food waste at retail and consumer level (some experts argued that in certain LMIC in regions the generation of food waste is very low and the majority of the groceries are bought in local markets that prioritise fresh, unprocessed foods). Hence, experts reasoned that some policies would apply best to contexts where the specific challenges associated are high.

The following key topics were raised several times during the discussions in the workshops: (1) the need to change the wording from ‘healthy and sustainable [crops/foods/diets]’ to ‘healthier, more sustainable [crops/foods/diets]’ to ensure a flexible, context-specific meaning; (2) the difficulty (due to the lack of scientific and empirical evidence) to understand the effects of some proposed policies that have never been implemented, particularly with regards to different contexts, settings and populations; (3) the urgency to address the gap in literature regarding women’s empowerment, and to understand which are the effective policies and the barriers to policy development; (4) the crucial role that proper design, implementation, monitoring and evaluation play in ensuring that the effect and effectiveness of the proposed policies is attained; (5) the need to differentiate according to national, regional or local jurisdictions when assessing the effects, effectiveness and potential synergies or trade-offs; and (6) the difficulty of determining which synergies or trade-offs may arise from each of the proposed policies, without being able to apply them in a specific context (as they may vary due to social, economic and environmental factors).

Some experts also suggested keeping the gender perspective as a cross-cutting topic across policies, as to ensure women’s social protection and recognition in all aspects related to the food system. In this vein, another suggestion was to adapt the proposed policies to make them gender-neutral (for instance, by including terms such as “farmers” or “fishers” instead of adding a disclaimer at the end of the policy such as “including women and vulnerable groups”), as a mechanism to avoid the misconception of having to include women as if they were a minority group. Additional concerns raised referred to ensuring that consumers could prioritise healthy, environmentally sustainable and fresh products, as some interventions in the subdomains of food composition, labelling or waste are only applicable to processed foods. From these discussions, we identified additional potential trade-offs that to the best of our knowledge have not yet been reported by the literature. For instance, with regard to food reformulation, experts were concerned about the impact that such policies would have on prices and their subsequent effect on vulnerable groups.

As the initial purpose was to have only policies with double- or triple-duty potential, a common suggestion made by experts was to keep some proposed policies that only impact one outcome, but that were very effective and valuable for the sustainability of food systems. Such four “important single-duty actions”, all part of the ‘food production’ subdomain, were kept in the final list: (1) sustainable carbon sequestration practices; (2) sustainable fisheries; (3) optimisation of water resources management; and (4) climate change impact preparedness. Also within the subdomain of food production, there were some common concerns regarding agriculture/food production. First off, experts expressed the need to differentiate between support or subsidies provided to farmers/businesses producing healthy and sustainable foods for human consumption, versus those producing healthy and sustainable foods for animal feed (i.e. corn, soya, oats). Secondly, experts from LMICs highlighted the importance of including livestock production with agroecological principles for countries where mixed farming, small-scale production, and rural/family farms are largely dominant, as there the sector remains critical to food and nutrition security. A third topic raised by experts was that the majority of support from the government in neo-liberalist economies tends to go to big companies, while there should be a switch towards supporting smallholder farmers, start-ups, and small and medium-sized enterprises (SMEs).

The complete feedback received from the workshops, the reasons for exclusion/inclusion, and the information analysed which led to final list of proposed policies is available in Annex 4.

### Prioritisation of proposed policies

Table 4 provides an overview of the results from the ranking, including the codes and titles from the 27 proposed policies for food supply chains, and the 17 for food environments.

**Table 4:** Titles of the proposed policies for creating sustainable food systems, ranked according to their perceived prioritization level by international experts

Proposed policies		
**Rank position**	**Food supply chains**	**Food environments**
1	Subsidies for sustainable healthy crops/livestock/fish	Affordability of healthier and more sustainable diets
2	Incentives for crop, fish and livestock diversification	Subsidies for healthier and more sustainable foods
3	Land use management	Front-of-pack nutrition/environmental labelling
4	Support for start-ups and small- and medium-sized enterprises producing healthier and more sustainable foods	Taxes on less healthy/sustainable foods
5	Regulation framework at the retail level	Marketing restrictions of less healthy and less sustainable foods to children across all media
6	Food loss and waste reduction through a step-wise process	Evidence-based claim regulations
7	Support for women's Empowerment	Reformulation of processed foods
8	Effective use of trade policy levers for sustainable food systems	Restriction of marketing of less healthy, less sustainable food in retail outlets
9	Regenerative agriculture	School food and nutrition policies
10	Food loss prevention and reduction through infrastructure investment	Accessibility of shorter food supply chains to consumers
11	Optimisation of water resources management	Public sector setting (other than school) food and nutrition policies
12	Farmers and fishers' support	Reformulation of out-of-home meals
13	Climate change impacts preparedness	Nutrition information panels and ingredient lists
14	Ecosystem restoration and conservation	Out-of-home eating outlets’ menu labelling
15	Environmental impact measures	Marketing restrictions on breastmilk substitutes
16	Risk impact assessment of trade and investment agreements	Zoning laws for healthier, more sustainable retail outlets
17	Farmers’ access to traditional seeds and breeds	Prominence of healthy, sustainable foods in the (in)formal food sector
18	Connecting smallholder farmers with territorial markets	
19	Sustainable carbon sequestration practices	
20	Evidence-based reduction of the use of fertilisers	
21	Evidence-based use of fortification programmes	
22	Sustainable fisheries	
23	Trade incentives for shorter food supply chains	
24	Evidence-based reduction of the use of pesticides	
25	Evidence-based use of bio-fortification programmes	
26	Support to young generations	
27	Transparency of global food supply chains	

A total of 21 Food-SAT and INFORMAS2.0 experts participated in the final meeting to discuss the final list of proposed policies. Ten experts completed the survey, resulting in 13 complete rankings: one for undernutrition, four for obesity/NCDs, six for environmental sustainability, and two for health inequalities. No rankings were completed for women’s empowerment. Therefore, the extent to which experts agreed on the level of priority of the proposed policies could only be calculated for the outcomes of ‘obesity/NCDs’ and ‘environmental sustainability’. Using the Gwet AC2 coefficient, the agreement among experts for ‘obesity/NCDs’ was moderate for the food supply chains policies (0.56), and fair for food environments policies (0.38). The agreement among experts for ‘environmental sustainability’ was moderate for food supply chains (0.58) and for food environments (0.58). The complete analyses for the ranking and the experts’ agreements are available in Annex 5.

From the top-five ranked food supply chains policies, two were perceived to have triple-duty potential: (a) incentives for crop, fish and livestock diversification, and (b) support for start-ups and small- and medium-sized enterprises (SMEs) producing healthier and more sustainable foods. The other three top-ranked policies had perceived double-duty potential (two for obesity/NCDs and environmental sustainability, and one for undernutrition and environmental sustainability). For food environments, three of the top-five had perceived triple-duty potential: (a) affordability of healthier and more sustainable diets, (b) subsidies for healthier and more sustainable foods, and (c) marketing restrictions of less healthy and less sustainable foods to children across all media. The other two top-ranked policies had perceived double-duty potential (both of them for obesity/NCDs and environmental sustainability).

## Discussion

This study set out to create a comprehensive list of policies to achieve healthier and more environmentally sustainable food systems for governments at any level of jurisdiction. From the compilation of 291 international policy recommendations, a preliminary list of proposed policies was created. The findings from the prior conducted scoping review [4] highlighted that some policies included in the list, once implemented by governments, have beneficial effects in multiple outcomes analysed (double- or triple-duty potential). However, not all the proposed policies have been designed or implemented to date, displaying some important gaps in the evidence available. Combining the evidence from the scoping review and the survey with experts, it became clear that there is a wealth of policies that can potentially help tackling the global Syndemic. Given the complexity of food systems, and the high heterogeneity of potential effects depending on the country and setting, many policy options have trade-offs that should be considered and tackled during the policy design phase. Bringing all the information together during the workshops with international experts, we recognised that sometimes they can be mitigated with proper policy design, implementation and monitoring. We also learned that, despite some differences in contexts (which should be carefully evaluated when designing/implementing the proposed policies), the challenges and needs within food systems are similar across the globe, with some population groups being forsaken when designing policies. Nevertheless, some outcomes are perceived as more important than others, depending on the context. The list of 44 proposed policies created as a result of these steps highlights the enormous potential of policies to improve the healthiness and sustainability of food systems worldwide. Based on the final ranking, we were able to identify which proposed policies experts perceive should be prioritised by governments.

The identification and prioritisation of specific policies to achieve healthier and more environmentally sustainable food systems remain a challenging task due to the number of variables at play, and the complex ways in which they interact with one another. As highlighted in the scoping review and mentioned by experts during the workshops, some proposed policies would need to be implemented in conjunction with others in order to achieve the highest possible benefit (for instance, marketing restrictions with FOPNL and fiscal measures). In addition, some proposed policies may be relevant only within certain contexts (such as water fountains in schools or food waste reduction mechanisms). Prior to their implementation, the proposed policies should be adapted or combined with others to take contextual factors into account and be able to obtain optimal results.

As shown in the ranking results, the policies perceived to have triple-duty potential did not always necessarily score higher in the prioritisation ranking when all the outcomes when considered. This was the case of some policies that have been scientifically proven to have triple-duty potential (i.e. school food and nutrition policies or regenerative agriculture) or which were perceived as such by experts (i.e. marketing restrictions of breastmilk substitutes; connecting smallholder farmers with territorial markets; prominence of healthy, sustainable foods in the (in)formal food sector) occupied lower positions in the ranking. In fact, out of the first five-ranked food supply chains policies, only two had been perceived to have triple-duty potential, namely the incentives for crop, fish and livestock diversification, and the support for start-ups and SMEs producing healthier and more sustainable foods. For food environments, three of the five had perceived triple-duty potential, which were affordability of healthier and more sustainable diets, subsidies for healthier and more sustainable foods, and marketing restrictions to children across all mediaOn the other hand, the “single-duty” policies that were advised to keep (even if their impact was only in one outcome) occupied higher positions in the ranking than others with perceived double- or even triple-duty potential. This was the case with mechanisms for optimisation for the management of water resources, climate change impact preparedness or sustainable carbon sequestration practices. These findings bring an interesting policy perspective to the concept of the Global Syndemic, showing whilst the effects of the policies across outcomes remain important during the prioritisation process of actions to undertake, other factors may alter their relevance and hierarchical decisions.

Based on the feedback from experts, this study identifies the potential double- or triple-duty, synergies and trade-offs from the policies that would otherwise be hard to analyse without empirical policy implementation. However, synergies and trade-offs are also context-specific and the policy effects will inevitably vary depending on social, economic and political factors. In complex systems, policy changes can have unintended or unexpected effects. Therefore, as shown by similar research in this field [5, 23], the synergies and trade-offs identified in this study should not be taken as a deterministic assessment of what would be certain to happen if the proposed policy is implemented. It should instead be interpreted as a potential scenario to consider during the agenda setting, policy design, implementation, or monitoring phases.

In order to be applicable in different jurisdictions and contexts, and based on the experts’ feedback, we decided to propose policies that are broad in scope. The regional workshops were particularly useful as they combined the knowledge from global experts (with experience in diverse settings) with the evidence available from the scoping review on the effects and effectiveness of policies that have been implemented. However, during the workshops and the meetings with experts prior to the ranking, there were some inevitable discrepancies in their views. For instance, experts in Latin America stressed the importance of the styles of FOPNL, as they argued that warning labels (such as those used in Chile or Mexico) have similar effects across population groups, whereas experts in Europe considered colour-coded label schemes to be more effective among higher-income groups. Other controversial topics were the use of biotechnology and genetically-modified organisms (GMOs), or the reduction of consumption of red and processed meats, as experts from Europe and Latin America had a different approach to this regard compared to experts from East and West Africa. These examples show that the proposed policies should not be implemented without a careful evaluation of their suitability to a specific context, and sound scientific evidence.

There are also many differences in perceived double- or triple-duty potential, or in the number of synergies and trade-offs identified when comparing the scientific with the perceived potential, given that during the scoping review we could not find data for each policy and outcome. Therefore, our results should be placed in context to ensure a correct interpretation of the potential effects of the policies. Effectively, by compiling a list of proposed policies, together with their perceived double- or triple-duty potential, and their identified synergies and trade-offs, we aim to make a strong case for applying a comprehensive and flexible approach toward food systems policy design/implementation. Nonetheless, it will require significant efforts from governments and food stakeholders worldwide, as food systems policies are highly interlinked to globalisation dynamics due to their diverse effects on population nutrition and environmental sustainability [27].

Some of the learnings from bringing together evidence with expert opinion are that they were sometimes able to identify additional effects that, to the best of our knowledge, are not available in the literature. For instance, during the workshops it was suggested that mandatory food reformulation strategies may increase the prices of those products and therefore make them less accessible to vulnerable groups, already at higher risk of developing obesity and diet-related NCDs [19, 21, 30].

The addition of synergies and trade-offs in our list provides a reminder to both academics and policymakers that successful efforts towards healthier and more environmentally sustainable food systems can both positively and negatively impact certain outcomes or population groups. For instance, when it comes to designing policies for food production, policymakers and researchers should focus on more than just food insecurity, and consider other factors (such as environmental sustainability, the diversity/variety and quality of diets, and the impact the policies may have on workers, linked to inequalities and women’s empowerment). In that line, as suggested during the workshops and surveys with experts, some actions considered to be environmentally sustainable may require farmers to employ more manual labour. As in many contexts, women provide most of the manual labour in agriculture, a larger share of labour-intensive tasks could affect their health and make it harder to achieve autonomy.

### Strengths and limitations

The current approach taken to develop the proposed policies to create healthier and more environmentally sustainable food systems has several strengths. Most importantly, the collaboration with experts across domains and regions ensured a holistic view of challenges and political solutions. Adding on to this, the inclusion of international reports, scientific literature as well as expert opinions made it possible to obtain novel insights and ensure the creation of a tool applicable within different contexts and across many levels of jurisdiction. In our opinion, a major strength of this research was the multi-country research collaboration, ensuring input from experts living in different settings.

Nonetheless, there were also several limitations, despite the fact that the methodology we followed to identify and combine all steps of this study aims to show transparency in how we developed the results. A limitation of this study is that, while all our sources approached food systems from a global perspective – and were authored by international researchers – several of the initially recommended policies were more relevant to higher-income countries, potentially reflecting a bias in the explicit recommendations made by the documents reviewed. This limitation was also reported in similar research collecting international policy recommendations [5]. Nevertheless, during the additional steps undertaken, and in particular through the regional workshops organised across different LMICs, we tried to address such bias. Another plausible limitation of this study is that, inevitably, experts did not always agree on some controversial topics (i.e. red meat reduction, use of biofortified foods), and it was not always clear which opinion should be the one taken into account. In order to solve this, we decided to include these topics despite their controversial aspect but to keep the scope broad for further context-specific considerations. Moreover, the fact that environmental sustainability includes such varied dimensions that sometimes even present trade-offs among themselves (such as GHGEs, biodiversity loss, freshwater use, and soil health), made it particularly hard for experts to accurately determine the effect of the policy on environmental sustainability. It is also important to highlight that the agreement levels among experts in the ranking were fair and moderate for the outcomes analysed, which may be seen as an additional limiting factor.

### Future steps

While our focus has been on assembling a list of proposed policies based on international reports, scientific literature, surveys, and workshops, we recognise that many gaps in the literature remain with regard to their effect, effectiveness and additional potential trade-offs. Nonetheless, our work provides a strong starting point for further reflection on how policies can be designed, and on which policies could be implemented. As future steps, it would be interesting for researchers to explore the levels of implementation of such policies in specific countries, regions, cities or municipalities, and to further analyse their potential effects, synergies and trade-offs through additional (quantitative and qualitative) studies. It would also be pertinent to explore which are the levels of jurisdiction of each of the policies according to countries, and the type of actors involved in their design, implementation, monitoring and evaluation. For policy-makers, the results of this study provide a holistic and transdisciplinary list of actions that can be consulted to increase synergies and avoid potential trade-offs when designing/implementing public policies, interventions and programmes to achieve healthier and more environmentally sustainable food systems.

## Conclusion

Based on our findings from two online surveys and a consultation process with international experts, and taking into account the results from a previously conducted scoping review, we created a list of 44 proposed policies for governments to achieve healthier and more environmentally sustainable food systems. Forty of the proposed policies are perceived to have double- and triple-duty potential to tackle the global Syndemic. The proposed list serves as a starting point for catalysing the needed change of global food systems. It is important to note that, to address all the complex aspects of food systems, the proposed policies should be contextualised and adapted to each situation and environment. Priority could be given to those policies/interventions with scientific, evidence-based effectiveness, and to those identified to have higher levels of prioritisation.

### Supplementary Information

Supplementary Material 1.

Supplementary Material 2.

Supplementary Material 3.

Supplementary Material 4.

Supplementary Material 5.

## Data Availability

All the data presented in this study are available upon reasonable request from the corresponding author.

## Footnotes

1 INFORMAS 2.0 IDRC project – available in: //www.idrc.ca/en/project/harmonized-indicators-measuring-progress-toward-more-sustainable-healthier-food-systems
