# Supplementary material for: Which government policies to create sustainable food systems have the potential to simultaneously address undernutrition, obesity and environmental sustainability?
Source: Global Health. 2024 Jul 27;20:56. doi: 10.1186/s12992-024-01060-w (PMC11282665; doi:10.1186/s12992-024-01060-w)
Supplement: Supplementary file 2 — Supplementary Material 2. [file 12992_2024_1060_MOESM2_ESM.docx]

**Annex 2.** Groups to which the 96 international experts were assigned during the regional workshops, and the total number of policies discussed at that stage (n=42). The division was done based on their field of expertise in the food subdomains identified.

| Groups | Subdomains discussed | N° of policies discussed *per subdomain*, (per group) | N° of experts  *per region*,  (per group) | Field of expertise | Country representation | Organisation type |
| --- | --- | --- | --- | --- | --- | --- |
| Group 1 | - Food production | *12*  (12) | *Europe: 6*  *Latin America: 4*  *Africa (EN): 9*  *Africa (FR): 5*  (24) | *Agriculture (24)* | *Belgium (1), Benin (1), Chile (1),  Colombia (2), Germany (1), Iceland (1),  Kenya (6), Mexico (1), Norway (1),  Poland (1), Senegal (2), Spain (1),  Tanzania (1), Togo (2), Uganda (2)* | *Academia (9)*  *NGO (6)*  *Public sector (9)* |
| Group 2 | - Food storage, processing, packaging and distribution - Food loss and waste - Food retail | *6*  *3*  *3*  (12) | *Europe: 4*  *Latin America: 4*  *Africa (EN): 9*  *Africa (FR): 6*  (23) | *Food processing (19)*  *Food storage and distribution (3)*  *Food waste (1)* | *Benin (1), Brazil (1), Burkina Faso (2), Colombia (1), Côte d’Ivoire (2), Ecuador (1), Germany (1), Italy (1), Kenya (6), Mexico (1),  Norway (2), Rwanda (1), Senegal (1),  Uganda (2)* | *Academia (9)*  *NGO (4)*  *Public sector (10)* |
| Group 3 | - Food trade and investment - Food prices | *3*  *3*  (6) | *Europe: 4*  *Latin America: 3*  *Africa (EN): 5*  *Africa (FR): 4*  (16) | *Food trade (4)*  *Food prices (12)* | *Argentina (1), Belgium (1), Benin (2),  Brazil (1), Germany (1), Kenya (2),  Malawi (2), Mexico (1), Poland (1),  Senegal (1), Togo (1), Uganda (1),  United Kingdom (1)* | *Academia (6)*  *NGO (4)*  *Public sector (6)* |
| Group 4 | - Food composition - Food labelling - Food promotion - Food provision | *2*  *4*  *3*  *3*  (12) | *Europe: 13*  *Latin America: 10*  *Africa (EN): 5*  *Africa (FR): 5*  (33) | *Food environments (33)* | *Argentina (2), Belgium (1), Benin (1),  Brazil (2), Burkina Faso (1), Chile (1),*  *Côte d’Ivoire (1), Ecuador (1), Estonia (1), France (1), Ghana (1),  Guatemala (1),Ireland (1), Italy (1), Jamaica (1), Kenya (2),  Mexico (2), Norway (1), Portugal (2),  Senegal (1), Spain (2), Tanzania (1),  The Netherlands (2), Togo (1),  Uganda (1), United Kingdom (1)* | *Academia (17)*  *NGO (9)*  *Public sector (7)* |
|  |  | **42** | **96** |  |  |  |

**Legend:** EN: English. FR: French. NGO: Non-governmental organization.
